# Maternal–Fetal Surgery: Does Recognising Fetal Patienthood Pose a Threat to Pregnant Women’s Autonomy?

**DOI:** 10.1007/s10728-021-00440-2

**Published:** 2021-10-21

**Authors:** Dunja Begović

**Affiliations:** Centre for Social Ethics and Policy, The University of Manchester, Williamson Building, Oxford Road, Manchester, M13 9PL England

**Keywords:** Fetal surgery, Maternal–fetal surgery, Maternal–fetal conflict, Autonomy, Pregnancy, Fetal patient

## Abstract

Maternal–fetal surgery (MFS) encompasses a range of innovative procedures aiming to treat fetal illnesses and anomalies during pregnancy. Their development and gradual introduction into healthcare raise important ethical issues concerning respect for pregnant women’s bodily integrity and autonomy. This paper asks what kind of ethical framework should be employed to best regulate the practice of MFS without eroding the hard-won rights of pregnant women. I examine some existing models conceptualising the relationship between a pregnant woman and the fetus to determine what kind of framework is the most adequate for MFS, and conclude that an ecosystem or maternal–fetal dyad model is best suited for upholding women’s autonomy. However, I suggest that an appropriate framework needs to incorporate some notion of fetal patienthood, albeit a very limited one, in order to be consistent with the views of healthcare providers and their pregnant patients. I argue that such an ethical framework is both theoretically sound and fundamentally respectful of women’s autonomy, and is thus best suited to protect women from coercion or undue paternalism when deciding whether to undergo MFS.

## Introduction

Despite the steady progress of prenatal diagnostic and testing procedures since the mid-twentieth century, prenatal *treatment* of the fetus is usually not an option upon diagnosis. Most women who receive a result indicating the presence of disease or disability in the fetus are faced with the choice of either terminating the pregnancy or preparing for the birth of a child with a medical condition [32]. The development of maternal–fetal surgery^1^ (MFS) introduces a third possibility: treatment that can alleviate, or even eliminate, fetal medical issues before birth. While these procedures are highly significant in helping avoid the dilemma between pregnancy termination and having a child with a disease or disability, they also bring in a host of critical ethical issues [19, 51]. As these treatments are further improved and introduced into medical care, it is necessary to consider what kind of ethical framework would best protect the interests of the pregnant woman, while also taking due regard for fetal welfare.

In this paper, I contribute to this discussion by examining some existing models of the maternal–fetal relationship in the context of MFS, evaluating their potential ethical implications and how they respond to the problem of maternal–fetal conflict. To do this I pose, and attempt to answer, two main questions: 1. Who is the patient in maternal–fetal surgery: the pregnant woman, the fetus, or both? 2. Does recognising fetal patienthood pose a threat to pregnant women’s autonomy in MFS? The goal is to propose an ethical framework that is conceptually sound and which, in its practical application, would ensure that pregnant women’s autonomy is respected when making the decision whether to engage in MFS. A sound conceptualisation of MFS is necessary to identify the right ethical questions for further exploration, and to serve as professional guidance that may impact outcomes for pregnant women and their fetuses. While the law must continue to recognise only one patient within the context of MFS, I will argue that there are important ethical reasons why there should be some very limited scope for recognising the fetal patient when considering its outcomes and implications.

## Maternal–fetal Surgery: an Emerging Development

MFS encompasses a range of innovative procedures aimed at treating fetal illnesses and anomalies during pregnancy. This involves accessing the fetus through the body of the pregnant woman and performing a surgical procedure on it, after which it is returned to the uterus until birth. Examples of conditions that can be treated with MFS include urinary tract obstructions, diaphragmatic hernia, and congenital lung lesions [7]. MFS procedures vary in their invasiveness, with three basic types of techniques in use: open surgery involving an incision into the uterus through the abdominal wall (hysterotomy); fetoscopic surgery which is a less invasive procedure with a smaller incision; and percutaneous fetal therapy delivered through a catheter [45].

One of the most prominent applications of MFS has been in the prenatal treatment of spina bifida,^2^ a neural tube defect occurring during pregnancy that can lead to issues such as paralysis of the legs, incontinence, hydrocephalus, and learning difficulties [42]. In the rest of the paper I will use this widely studied example of MFS as a template for discussing the ethical concerns arising in the general context of prenatal surgical interventions. The standard treatment for spina bifida used to be postnatal surgical intervention. However, studies, such as The Management of Myelomeningocele Study (MOMS) in the US, demonstrated that prenatal surgery for spina bifida performed before 26 weeks gestation has an overall positive effect on preserving neurological functions in the fetus and reversing existing anomalies [1, 2]. The surgical technique used for this treatment is typically the invasive, open surgery variant of MFS [3, 39]. In recent years there has been a move towards using fetoscopic techniques, but further clinical trials are required to determine their efficacy [36], and there is currently a lack of standardised procedures and high-quality evidence to ascertain their purported advantages over the open surgical procedure [54].

In the UK,^3^ several successful prenatal interventions for spina bifida have been conducted in recent years [8, 9]. In August 2018, NHS (National Health Service) England opened a public consultation on providing MFS for spina bifida in select medical centres [41], and NICE (National Institute for Health and Care Excellence) announced the beginning of funding for open surgical repair in September 2019 [44]. At the time of writing, an MFS programme for open spina bifida surgery is available on the NHS through two Fetal Surgery Centres [43], and as of May 2021, 32 MFS procedures have been successfully performed as part of a collaboration between British and Belgian institutions, even throughout the COVID-19 pandemic [21]. All procedures in the UK were done using the traditional open surgery variant, while the less invasive, fetoscopic variant is currently only considered as a research procedure [53].

Alongside promising clinical outcomes and the possibility that surgical techniques could address a broader range of medical conditions prenatally, many important ethical and practical issues arise in the context of MFS. An evaluation of the MOMS trial outcomes acknowledged that the procedure carries non-negligible risks of adverse effects on the health of both pregnant women and fetuses, suggesting that the surgical technique needs to be improved [1]. At this point, both the open surgery and fetoscopic variant of MFS entail potential harms to the pregnant woman [54], though the more invasive variant has been shown to pose significantly higher risks [29]. There are also risks to fetal outcomes and the remainder of the pregnancy. Physical damage can result to both the pregnant woman and the fetus, and the woman may also incur psychological harm, for instance when the procedure is not successful. Other important issues include the costs and availability of these innovative procedures, as well as broader issues of research ethics and responsible innovation [19].

So-called maternal–fetal conflict, occurring when clinicians have conflicting obligations to the maternal and fetal patient respectively, is often seen as the main ethical issue in MFS [17]. However, the very plausibility of such a conflict occurring depends on whether we accept that the fetus can be a patient in the first place. To this end, I will now examine some existing models of the maternal–fetal relationship in MFS (in terms of who is considered to be a patient), and examine the arguments in favour of and against each.

## Who is the Patient in Maternal–fetal Surgery?

Determining the obligations owed to different parties in MFS requires first answering the question who or what exactly counts as a patient in this context. Two basic conceptualisations of the maternal–fetal relationship in the existing literature are the one-patient and two-patient model [30]. On the former, the pregnant woman is considered to be the sole patient, with the fetus fully dependent on her for its survival and development.^4^ Adopting this model would mean that we need only consider the pregnant woman’s decision about whether to engage in MFS. On the latter model, the fetus is recognised as a patient in its own right that can potentially have clinical interests distinct from those of the woman. The consequence of adopting this model could be a need to evaluate the interests of both patients when making decisions about MFS. Besides these basic two models, there are also some more sophisticated accounts that seek to overcome the tension between them, but that could be said to still fall somewhere in between these two camps.

### One or Two Patients?

Some scholars, both in ethics and law, take the position that the fetus should be treated as part of the mother for the entire duration of the pregnancy up until birth, in which case there would be only one patient. This is also the approach adopted by many jurisdictions, such as the member countries of the European Union and the United States. In English law, some prominent court decisions have established that the fetus cannot be afforded legal personhood or any rights following from this: of particular significance are the cases of *Paton v British Pregnancy Advisory Service*, in which a husband unsuccessfully attempted to prevent the termination of his unborn child citing a right to life [12]; and *Re F. (*in utero*)*, in which a local authority was denied their application to make the unborn child of a patient a ward of the court because the fetus has no status or rights of personhood in law allowing such an intervention [40].

The one-patient model has the distinct advantage of being consistent with how the law generally regards patienthood in MFS. Besides consistency with the law, there are also strong ethical arguments for treating the pregnant woman as the only patient. A significant worry about conceptualising the fetus as a patient is that this will lead to women being sidelined when debating the ethical implications of MFS, as the fetus might eventually come to be seen as the primary patient, or the more important of the two [34, 51]. Also, it seems conceptually and practically clear what we mean by saying that the pregnant woman is a patient in MFS. But it is less clear what it would mean for a fetus to be a patient, leading us to examine the two-patient conception of MFS.

It seems uncontroversial that pregnant women are patients in MFS, as their bodies are directly involved, and they are autonomous agents who are owed a duty of care. Fetuses certainly lack this key feature of autonomy. However, autonomy may not be *necessary for patienthood*, as we routinely speak of newborn babies or people in comas as patients, even though they are not autonomous. The fetus however, or more accurately the future child, stands to benefit directly from the surgical procedure, which cannot be said of the pregnant woman (except perhaps psychologically). When a newborn baby is treated for an illness, despite its complete lack of a developed sense of self, it seems plausible to speak of its clinical interests and the effects of treatment on its well-being. There is a significant (some would say crucial) difference in that the fetus exists *within* the woman’s body, so it depends on the mother for its life in the most literal sense. Still, if women are ready to permit interventions on their body in order to access the fetus and perform surgery on it, would clinicians be justified in treating that fetus as a kind of temporary patient [48]?

The two-patient view is often encountered in both public perceptions and the scientific literature, especially clinical [for some examples see 31, 46], but also ethical, as in this recent definition: “Surgical intervention *on behalf of a fetus* takes place, of course, inside a pregnant woman’s body, hence the reason it is sometimes called maternal–fetal surgery. (…) The ethical issues of fetal surgery are complicated since any intervention is invasive, often experimental, and *involves two patients*.” [24, my emphasis] This definition clearly shows in which sense the fetus might be seen not only as *a* patient, but as the *primary* patient, despite acknowledgment that it is confined to the woman’s body and that these interventions may pose risks to her health and well-being. Such framing fuels some of the worries about recognising fetal patienthood that will be discussed later in this paper.

Something like a middle ground between the one- and two-patient models can be found in accounts that take as a starting point the biological interdependence of the pregnant woman and fetus, and then propose a sense of patienthood that remains accepting of and appropriate to this basic fact. One such account is the ‘two-patient ecosystem model’ advocated by Susan Mattingly [37]. This model of the maternal–fetal relationship stresses the biological unity and inseparability of the dyad. The fact that the fetus is *incorporated* in the pregnant woman in a very literal sense, on her account, will always trump any conceptual differentiation between the two. Mattingly suggests that it is precisely equivocation between the one- and two-patient accounts that leads to conceptual and practical confusion: “(…)treating the fetus as an independent patient but continuing to regard the pregnant woman as a compound patient incorporating fetus–has, I think, caused the physician's ethical dilemma to be misconstrued as a conflict between the duty to benefit the fetus and the duty to respect the woman's autonomy. (…) But fetal therapy is beneficial to the pregnant woman only on the old model, where she *includes* the fetus, while fetal harm is harm to another only on the new model, where the fetus is independent and exclusive of the woman.” [37, emphasis in original] According to her analysis, furthermore, recognising the fetus as a distinct patient actually strengthens the obligations doctors have towards pregnant patients: “Ironically, when the fetus is construed as a second independent patient, physicians' prerogatives to act as fetal advocates are actually diminished. This consequence flows not from any assumed superiority of maternal rights over fetal rights, but from differential professional duties to donors and recipients of medical benefits.” [37] Mattingly ultimately suggests that the way forward is not to deny the possibility of fetal patienthood, but instead “challenge the orthodox view of the professional-patient relationship, which suppresses dependency relations among patients and posits them as strangers to one another” [37], suggesting a family-oriented model of illness and treatment which focuses on relationships, protection, dependence and care.

This account seems highly plausible, not only in how it characterises the maternal–fetal relationship starting from the biological nature of the unit, but also in its demonstration that both the standard one-patient and two-patient models are respectively too simplistic to successfully address the ethical and practical reality of medical decision-making in pregnancy. However, its immediate normative implications, especially for the MFS context, are not entirely clear. The ultimate conclusion Mattingly reaches seems to be that maternal and fetal patients should be treated together almost as a compound patient: “when the various possible models of the maternal–fetal dyad are consistently applied, they converge to reinforce the physician's customary ethical stance—working cooperatively with the pregnant woman for common, linked goals of infant, maternal, and family well-being.” [37] This certainly should be, and seems to be, the usual goal of maternal–fetal specialists involved in MFS [5], but some would argue that wherever we have two patients, there is also the potential for conflict between their interests—and thus a way to resolve such conflicts is needed. I will now discuss an account which attempts to address precisely this problem, in various clinical contexts including MFS and prenatal therapy.

### The Case for a Limited Sense of Fetal Patienthood: Chervenak and McCullough’s Ethical Framework

The standard two-patient model of the maternal–fetal unit in MFS posits that the fetus and the pregnant woman are two separate patients whose interests may conflict, leading to a situation where doctors may need to balance their obligations to each. This potential for separation can be addressed by stressing the unity of the maternal–fetal dyad, as Mattingly has done. Others, however, have suggested that a concept of fetal patienthood is necessary in order to resolve potential conflicts occurring, despite recognising that maternal and fetal interests are interwoven and independent [17]. A notable account is the ‘fetus as a patient’ framework developed by obstetric ethicists Chervenak and McCullough. On this conception, a human being becomes a patient when 1) it is presented to the physician for medical care, and 2) there exist clinical interventions that are “are reliably expected to result in a greater balance of clinical benefits over harms for the human being in question”. [14–16]. Whether a fetus is a patient, then, does not depend on whether it possesses sentience, personhood, or some kind of intrinsic value; instead, this is determined in relation to the physician’s ability to provide treatment and, crucially, by the pregnant woman’s choice to present it for care. This assigns the fetus a kind of *dependent* moral status, distinct from the one possessed by the pregnant woman on the grounds of her personhood-relevant characteristics, and stemming wholly from its position as a patient, which is established when the criteria stated above are satisfied.

There are several advantages to this framework. As mentioned above, it aims to keep the thorny discussion about the moral status of the fetus outside of the MFS debate, as the fetus is assigned only dependent moral status *as a patient*. This assignment is meant to shift the focus from the moral status of the fetus to the obligations owed to the respective patients by medical professionals, which is highly significant for practical purposes, especially for resolving situations of maternal–fetal conflict. This framework also assigns a crucial role to the pregnant woman, in that it is only her autonomous decision that can confer patient status upon the fetus. Despite positing the existence of a fetal patient, then, this framework also shows due regard for women’s autonomy by specifying that it is the pregnant woman who enables patienthood, and therefore dependent moral status, to be conferred upon the fetus by choosing to present it for treatment – at least up to a certain point, as they accept that the moral situation may change after the viability threshold is passed [14, 15]. The doctor is seen to have only beneficence-based obligations to the fetal patient, while having both beneficence- and autonomy-based obligations to the pregnant woman, because of the aforementioned differences in their moral status.

Chervenak and McCullough’s framework however also faces some pressing problems. Firstly, it focuses mainly on the application of the four-principles framework to balancing the differing obligations to the maternal and fetal patient in cases of conflict. While a highly influential bioethical account in its own right, the four-principles theory of Beauchamp and Childress has also faced various kinds of criticism, leading some authors to argue that Chervenak and McCullough’s application of the framework here inherits some of these potential problems [47]. Due to the prima facie nature of the principles involved (autonomy and beneficence), and lack of clarity on how these should be balanced in potential cases of conflict, these authors argue that the fetus as a patient account does not provide sound guidance for solving these problems in practice.

Secondly, significant objections are based on the possible threats to women’s autonomy and bodily integrity that may result from recognising the fetus as a patient. Chervenak and McCullough explicitly argue that, while the pregnant woman and the fetus are two patients, they are also inseparable and need to be considered together even if the respective obligations to each might differ. However, the worry here is that even acknowledging that the fetus and the pregnant woman might have separate, sometimes even conflicting interests, could justify *treating them as separate*, in the sense of acting in a way that will inevitably respect one set of interests but not the other [33, 34].

This ties into the final concern of whether their account truly manages to avoid granting independent moral status to the fetus. Rodrigues et al. point out that the conceptual connection between the fetus and the future child it will become actually smuggles in some kind of independent value for the fetus [47]. Moreover, it is worth highlighting that the primacy of the pregnant woman – her ability to confer moral significance onto the fetus and waive it at any point – only applies before the viability threshold is reached, even though the fetus remains within her body after that. Therefore, it seems that, despite acknowledging the centrality of women’s autonomy in making decisions MFS and developing a notion of patienthood that avoids assigning a fetus independent moral status, Chervenak and McCullough’s theory ultimately inherits some of the most pervasive problems of the traditional two-patient model.

The two slightly modified accounts (the fetus as a patient and the ecosystem model) present improvements in that they look at the actual, lived nature of the maternal–fetal relationship as a basis for their ethical position, and also in that they probe more deeply into the concept of patienthood instead of assuming that is has a clear meaning. Still, both of these views entail some recognition of fetal patienthood, therefore making it necessary to examine whether there are other inherent dangers to accepting the existence of fetal patients in any sense.

## Arguments for and Against Accepting Fetal Patienthood

Clinicians arguably see themselves as having fetal patients, as evidenced by their interest in developing new surgical procedures to treat fetal anomalies [26] and demonstrated views of priorities when conducting the procedures [5]. Of course, this in itself is not a convincing argument for accepting the existence of fetal patients. The professional and personal interests of clinicians leading them to explore MFS may not necessarily be ethically grounded or justified [51]. Also, while medical research and practice might recognise fetal patients, the law may not align with this, making it impossible for pregnant women to be compelled to submit to any medical procedure for fetal benefit. On the other hand, if we recognised the fetus as a patient in its own right, this could lead to attempts at regulating pregnant women’s behaviour to protect the fetus from harm. One reason to be sceptical of the two-patient framework thus is its divergence from existing legal standards, as they can be argued to be well-reasoned [38].

In contrast to the legal position, in the practical and medical setting the terminology used in medical writing and in newspaper articles about the technology indicates that the fetus is often considered to be a patient in MFS (see previous examples from recent BBC reporting, using language of ‘unborn babies’ having surgery in utero). There seems to be a tension between how MFS is regarded in legal and ethical debates, and how it is perceived by the medical profession and presented to the public. This may create a confusing situation for those directly involved in the process, namely pregnant women, their partners and healthcare providers, as well as those trying to formulate ethical guidelines for the practice.

This is evident already from the terminology used to describe prenatal surgical procedures: in earlier writing on the subject, and indeed some more contemporary literature, the most commonly used term for these surgical interventions was ‘fetal surgery’. Some have argued that such language obscures the fact that these procedures involve a direct, potentially risky intervention on the pregnant woman’s body [13, 28, 34]. Such arguments may have contributed to a shift towards referring to these procedures increasingly as ‘maternal–fetal surgery’, at least in scholarship on the topic. While being mindful about the language used likely raises awareness of the crucial role of pregnant women in the process, it is not clear that this change in academic terminology has wide-ranging implications for the public perception of MFS. News reports about MFS still use phrases like ‘surgery on unborn babies’ or ‘fetal repair’. This presentation of the issue suggests that, in the public eye at least, the focus is still very much on the fetus, while the risks of MFS to the health of pregnant women often go unmentioned.

A related objection is that recognising fetal patienthood could lead to women’s interests being perceived as secondary to those of the fetus, or even ignored altogether. Lyerly et al. argue that seeing the fetus as a patient could lead to doctors seeing it as a separate patient, with interests separate from those of the pregnant woman. This, in turn, could lead to disregarding women’s interests and their ‘erasure’ from MFS, not only symbolically but also practically. There is also the concern that women will face pressure from physicians, or members of their family and social circle, to undergo these procedures, possibly at significant risk to their own health and wellbeing [33, 34]. This is a highly important issue keeping in mind that harmful effects on women still accompany breakthroughs in MFS research [1, 2].

On the other hand, some also worry that women may, in certain cases, be prevented from going through with MFS in an unjustly paternalist way. If women are barred from pursuing MFS, despite feeling that this would be in the best interest of themselves and their future child, this may devalue their autonomy in the same way as expecting them to commit to extreme sacrifices for its well-being [51]. In either case, the worry is that women’s agency will be sidelined, thus diminishing their ability to make autonomous choices about what will happen in the course of their pregnancy. It is therefore suggested that healthcare policy and practice should adopt an approach in which the pregnant woman is considered the sole patient whose autonomous choices and interests must be taken into account.

Any sound ethical framework for MFS should aim to prevent women from being pressured into the surgical procedure if they do not want it. However, I would argue that an acceptable model must also account for cases in which women wish to do something that might not be in their best interests physically, even potentially affecting their health and well-being in the long term. After all, pregnancy itself involves various risks to women’s health, such as illnesses triggered by pregnancy, complications of childbirth, and postpartum depression. Yet pregnant women are frequently willing to take on these risks in order to bring a child to life when it is what they want. An ethical framework that truly respects pregnant women’s autonomy must acknowledge this potentially uncomfortable fact at its foundation. Thus in order to properly center women’s agency and experience in the ethical analysis of MFS, it is also essential to consider how pregnant women seeking or undergoing MFS actually perceive the procedure, their role within it, and the status of the fetus.

### The Views of Pregnant Women and Their Partners Partaking in MFS

Some qualitative studies conducted in recent years suggest that pregnant women involved in, or considering the possibility of undergoing, MFS or other types of prenatal therapy typically see the fetus as an ethically relevant entity, if not a person in its own right [23, 50], and are motivated to undergo such procedures by a desire to help the fetus, and thus their future child, have ‘the best life it can’ [6, 10, 18, 20]. A few primary themes emerge in these studies. Firstly, many of them report consistent usage of the words ‘child’ or ‘baby’ to refer to the fetus by pregnant women and their partners, suggesting that the fetus is perceived as a being in its own right, rather than as property or part of the woman’s body. One of the studies explicitly asked the participants who they consider to be a patient in the context of prenatal therapy, and several expressed the opinion that the pregnant woman was not the sole patient, with just over half stating that they believe the unborn child’s interests to be more important than the interests of its mother [23]. Secondly, an explicit commitment on part of the parents to ‘do everything they can’ [10, 18, 20] to help is frequently made, again implying that women and their partners perceive the fetus as having some interests of its own, or at least find it plausible to assess its well-being separately from their own. A majority of the participants in one study stated that they found it morally acceptable for a pregnant woman to submit to treatment for fetal benefit [23]. Finally, the themes of retaining hope despite the uncertainty that the procedure will yield a positive outcome, and being prepared for sacrifices to ensure the future child’s better health (out of a sense of parental responsibility), are very prominent in the narratives of those interviewed [6, 18].

So far a small number of these empirical studies have been conducted, and their conclusions have some limitations. The study samples are relatively small and obviously biased towards parents who are interested in the procedure. Also, since both MFS and empirical studies of it have so far been conducted only in a handful of countries, the views recorded in them are likely shaped by Western notions of pregnancy and parenthood, especially maternal responsibility towards the unborn child, which in turn have developed under the influence of new technologies [13, 25, 27]. One of the studies [10] features many of the participants using explicitly religious language in explaining their motivation to have the surgery, such as ‘being on a journey’ or ‘being tested by God’. Such views may not reflect how the majority of women perceive MFS or pregnancy in general.

Despite these limitations, however, the studies arguably put forth as a plausible hypothesis that seeing the fetus as a central figure in MFS is not limited only to the views of medical professionals, but is also prevalent in the accounts given by pregnant women and their partners. Such findings, along with the generally observed willingness of pregnant women to risk their health and welfare for the sake of the future child, imply that they perceive the procedure as not performed (solely) for their benefit, and indeed as not performed only *on them*. These findings suggest that the main actors in the process indeed perceive the fetus as a patient, at least in the temporary and specific context of the surgery being performed, which seems like something that should be acknowledged in ethical discourse and guidance on these procedures. In the rest of this paper I deal with what is probably the strongest objection to recognising any notion of fetal patienthood, namely, that this would lead to encouraging dangerous and pervasive views about maternal–fetal conflict.

## Does Accepting Fetal Patienthood Encourage the Conflict Framing of Pregnancy?

Maternal–fetal conflict is thought to arise when pregnant women behave in ways that are potentially harmful to their fetuses, for instance drinking or smoking excessively during pregnancy, or not adhering to doctors’ guidelines [11, 35, 52]. In the context of MFS, such conflict would occur if a woman refused to undergo surgery despite clear prospects for fetal benefit. In this situation, the beneficence-based obligations of physicians towards the fetus would clash with their beneficence- and autonomy-based obligations to the pregnant woman [14]. The two-patient model, as mentioned previously, is often criticised for obscuring the interests and thus jeopardising the autonomy of pregnant women in MFS. This infringement of autonomy would be most likely to occur in cases of maternal–fetal conflict, as on this model we might sometimes conclude that we should disregard pregnant women’s autonomous choices to ensure fetal benefit, for example by compelling them to undergo the surgery against their wishes, or using the interests and well-being of the fetus as a potential argument in getting a pregnant woman to reconsider or change their mind about engaging in MFS.

On the other hand, it is difficult to see how a maternal–fetal conflict could even occur on the one-patient model. If we consider the pregnant woman to be the only patient in MFS then it is clear that only her interests should be considered. This model does *not* imply that the fetus is entirely irrelevant: instead, the idea is that the best way to support fetal well-being is by supporting pregnant women’s choices [11, 22, 35]. This conclusion also seems to follow from adopting a dyad model. Mattingly proposes that we must focus on the biological unity of the dyad rather than focusing on the question of how many patients there are, as “literally, if not conceptually, the pregnant woman incorporates the fetus, so direct medical access to the fetal patient is as remote as ever” [37].

The notion of maternal–fetal conflict in general has been criticised for the inherent antagonism it posits within the maternal–fetal relationship, and its problematic assumptions about pregnant women’s motivations and agency. It has thus been suggested that this conflict framing of pregnancy ought to be abandoned completely [11, 22, 49]. Adopting the one-patient model for MFS then seems to be both a plausible and elegant solution: the woman’s autonomy is firmly centred within the ethical framework, and the focus on conflict is replaced by a commitment to ensuring that pregnant women considering MFS are provided with the best possible care and support in their decision-making.

While there are good reasons to reject the conflict framing of pregnancy in general, and accordingly adopt a one-patient model, I would suggest that the specific context of MFS presents some particular challenges. Despite significant progress in the last few decades, it still involves substantial risks to pregnant women, especially when more invasive surgical techniques are used (and these are still the dominant ones). The consequences for women’s health can be long-term and impact personal well-being and future pregnancies [1]. Arguably these risks may be ameliorated as the technology develops further. However, as the positive effects of MFS become increasingly visible, it is only to be expected that pregnant women will feel an even more pressing need to ‘do something’, or even ‘do anything’, to ensure the well-being of the fetus and thus their future child. It seems plausible that the current situation in which the risks of the procedure to women are not yet eliminated, but the fetal benefits seem too high to refuse, might remain the status quo for some time yet.

There are important reasons why the law should remain firm in treating MFS as it would any other potential intervention in pregnancy. The autonomy of pregnant women must be legally upheld against any recognition of fetal rights that could lead to the violation of pregnant women’s bodily integrity [38, 48]. However, I would argue that the ethical position on this issue can be more nuanced, to the extent that it may even diverge from the law at times. A sound ethical framework must take into account the particular moral situation at stake in MFS, and therefore there is a pressing need to develop an ethical framework which recognises the ethically relevant implications of the perspectives of pregnant women and medical professionals as participants in the process. Such an ethical framework is best placed to safeguard the autonomy of pregnant women, while giving due regard both to fetal clinical interests and women’s interests in benefitting their future children.

Yet, simply saying that ethical accounts may need to be more nuanced than legal solutions does not seem like a very satisfactory solution. As we have seen so far, the problems with recognising fetal patienthood of any kind are numerous and pervasive, so any attempt at resolving this tricky moral issue will have to say something about this. In the remainder of the paper I will try to sketch out a way of understanding patienthood that does not lead to the problems associated with fetal personhood, and to show why such an understanding would be useful for the ethical context of MFS.

## A Potential way Forward: Fetal Patienthood Without the ‘Threat’ of Personhood?

While several valid criticisms have been levelled at the Chervenak and McCullough ‘fetus as a patient’ account, I would nevertheless argue that their central intuition—that the fetal ‘patient’ in MFS should be treated as having a degree of ethical significance – seems plausible. It is clearly in this context an entity that warrants attention, conscientiousness and care from both the clinicians involved in treatment and prospective parents – and this view also seems to align well with their perceptions and experiences, as noted in the qualitative literature cited above. But there are also clear problems with ascribing moral status to the fetus at any stage that could have overarching consequences for the ethics of abortion and women’s autonomy in pregnancy. The central claim to address here, then, seems to be that the purported moral significance of the fetal patient in MFS also implies moral status, and thus rights, for the fetus.

Although it appears intuitively plausible, this argument seems to follow a strange trajectory. Normally, in arguments ascribing some kind of moral status or rights to the fetus, we would being by arguing that the fetus has some characteristic x (such as sentience, (potential) personhood, being a human, etc.) that gives it moral significance, therefore grounding rights and corresponding protections. But it seems to me that on this view it is argued that such moral significance would actually proceed from the fetus being *treated as* a patient, being subject to care and treatment.

The question of who counts as a patient and why is much too broad to address here, although I will suggest that this is something we need to gain a better understanding of for this debate. I would argue however that, generally speaking, ‘ordinary’ patients (namely competent adult humans) have rights, interests to be respected, duties owed to them etc. not because they are patients, but because they are *persons*. This is because all patient rights are fundamentally grounded in some variant of respect for autonomy and the informed choice of the individual – otherwise there would be no requirement of competence or capacity. Those patients that are arguably, according to some theories at least, not persons (e.g. people in persistent vegetative states, or anencephalic infants) are still considered to deserve treatment that respects their dignity – but dignity as a concept is often linked to some kind of respect for humankind, or avoiding unnecessary suffering, again linking to personhood-like criteria (even if fulfilled only partially or potentially). Alternatively, others may advocate for such patients and demand respect for what they believe would have been their autonomous choices (for instance, family members). Legally speaking, at least in the UK, patient rights also stem from their legal personhood.

Because legal personhood is a more clear-cut concept, it is easier to understand why a fetus could not be a patient legally speaking than in the philosophical debate (though, as some have noted, this may eventually change with the development of new technologies [48]). Nevertheless, in both domains, patienthood (understood as the holding of patient rights) is grounded in personhood – in possessing those traits that would satisfy the legal (or philosophical) criteria for someone to be a person. Because the moral status of the fetus is still a raging and unresolved debate, some, like Chervenak and McCullough, wish to avoid it altogether. But the sense of being a patient that they discuss still seems to be about there being certain obligations that a doctor has toward you. Is it possible to be a patient in any other sense?

I would argue that it is indeed possible for the fetus to be treated as a patient without having patient rights, and so without being a patient in the traditional sense. This is because we already have a ‘traditional’ patient – the pregnant woman – and the fetus can only receive treatment at her request, and with her consent. The reason doctors are able to successfully operate on fetuses is their anatomical similarity to newborn babies, and it is even possible that when performing such procedures they think of the fetus as a future child that they want to secure the best possible outcomes for. But this does not make the fetus a patient in the same way as a newborn or grown adult, as it does not have the corresponding rights or agency.

Rather than trying to keep the moral status of the fetus out of this debate, then, we should be clear in our attempts to grapple with this ethically that we do not take fetuses to be persons. It has been shown why fetuses being taken to have rights independent of the pregnant woman, and the resulting problem of maternal–fetal conflict, is both legally and ethically speaking a potentially dangerous idea (Romanis et al.), and further work is necessary to establish the *ethical* boundary between fetuses and born-alive, fully formed humans. But when it comes to ethically assessing MFS, I argue that ignoring or sidestepping the intuitions of those most directly involved in these procedures – pregnant women and their doctors – is not a solution for the discomfort we feel about the possibility of fetuses being recognised as morally significant entities in certain contexts. We must make it clear that just because the fetus is something that can be treated as a patient does not mean it will therefore attain the rights patients normally have. Furthermore, it should always be stressed that the fetus becomes a patient only by extension on the mother’s request—it is not a potential patient waiting to happen.

There are those who would argue that it is very difficult, or perhaps futile, to attempt to reconcile intuitions about fetal significance in MFS with upholding the convictions that fetuses are not persons and cannot have rights. Social science approaches track the shifts in how the fetus is perceived and engaged with, showing how the fetal patient is gradually constructed through interactions and changing narratives [13, 55]. It is becoming increasingly accepted that neither personhood nor patienthood are static categories, and that they develop along with social practices and technological advances [4]. In this article I have attempted to suggest such a compromise by rejecting both the simple one-patient and two-patient models in favour of a more sophisticated account that takes seriously the potentially problematic implications on both sides of the debate. Further argumentation about the ethical criteria for personhood and the concept of patienthood itself seem to me necessary to advance the bioethical debate; but for our present purposes, there is hopefully some use in acknowledging the difficulties that come with settling on overly simplified solutions.

## Conclusion

The primary ways of understanding the maternal–fetal relationship in ethical literature are the one-patient and two-patient model. I demonstrate that when applied to MFS neither of these withstands scrutiny, and suggest that an ecosystem-type model would be best suited to uphold pregnant women’s autonomy. Yet I argue that to any sound ethical framework will need to incorporate some limited notion of fetal patienthood. We should be cautious about centering the fetus as this kind of discourse can easily slip into talk of fetal rights and conflicting interests between pregnant women and fetuses. But we also need to be mindful of how the actual participants involved in the procedure perceive this situation. For that reason, I would argue that the tension between the ethics and the law here might be unavoidable, and that it is not necessarily a tension that we should seek to avoid at all costs. The law must stay firmly on the woman’s side as we do not want to recognise any form of fetal personhood for well-known reasons of upholding autonomy. But ethical discourse and the clinical guidelines it may influence should be open to the possibility of fetal patienthood in a limited sense, while also being sensitive to the developing state of MFS and the ethical implications of future advances in the technology.

Keeping in mind the current state of MFS, as well as public perceptions of the procedure and the reported motivations of participating parents, it is likely that there will be a growing emphasis on this option, encouraging its uptake in larger numbers. There is a pressing need for a strong ethical framework to inform everyday practice, especially as MFS enters mainstream healthcare and becomes more widely available, while the associated health risks of the procedure remain. Such an ethical approach must ensure the affirmation of pregnant women’s autonomy whether they wish to present their fetus as a patient or not, with the law acting as a safeguard to prevent any coercion from occurring. One of the first steps in constructing such an approach must be the recognition that pregnant women and clinicians are likely to see and treat fetuses as patients, and open up a broader conversation about the meaning of patienthood beyond a framework of respect for individual rights.

## Footnotes

1 In this paper I will use the term ‘maternal–fetal surgery’ to refer to these procedures. As noted below, the term ‘fetal surgery’ used to be more common, especially in medical literature. However, in the ethical debate there has been a move towards using the terminology of ‘maternal–fetal surgery’ in order to emphasise the point that the pregnant woman is also an important actor in this process. As this paper starts from the assumption that the pregnant woman is definitely a patient, and then questions whether the fetus can also be defined as one, I find this term to be the most appropriate for my discussion.

2 More precisely, MFS has been used to treat a severe variant of spina bifida called myelomeningocele, in which a larger portion of the spinal canal is exposed. However, the surgical procedure is usually referred to simply as MFS for spina bifida.

3 In this paper I will focus on the legal and practical context of England and Wales, albeit informed by scholarship and research from other countries, in particular the US. I hope however that the conclusions reached in the paper have broader ethical implications that could potentially be applied to other contexts and jurisdictions.

4 There are different ways of conceptualising this dependence. Some consider the fetus to be a body part or organ of the pregnant woman, while others view it as a sort of bodily property. A detailed discussion of the metaphysics of pregnancy however is beyond the scope of this paper.
